# Progression of idiopathic thoracic or thoracolumbar scoliosis and pelvic obliquity in adolescent patients with and without limb length discrepancy

**DOI:** 10.1186/s13013-018-0166-y

**Published:** 2018-09-24

**Authors:** Avraam Ploumis, Vikas Trivedi, Jae-Hyuk Shin, Kirkham B. Wood, Brian E. Grottkau

**Affiliations:** 1 Department of Orthopaedics, Spine and Paediatric Service, Massachussets General Hospital, Harvard University, Boston, MA USA; 2 Faculty of Medicine, Division of Surgery, Department of Physical Medicine and Rehabilitation, University of Ioannina, 45110 Ioannina, Greece

**Keywords:** Spinal curve, Anisomelia, Tilt

## Abstract

**Βackground:**

Both limb length inequality and scoliosis are associated with pelvic obliquity.

**Methods:**

This is an observational study of adolescents with growth potential presenting for evaluation of thoracic or thoracolumbar idiopathic scoliosis at an outpatient pediatric orthopedic clinic. Patients were evaluated for limb length discrepancy (LLD) (using bilateral femoral head height difference), pelvic obliquity (using bilateral iliac crest height difference and sacral takeoff angle), and scoliotic curve (using Cobb angle and rotation) on full spine standing radiographs. The same radiographic parameters were measured at a follow-up visit at least 2 years later.

**Results:**

Seventy-three consecutive patients with a mean (SD) age of 13.3 (0.2) years at initial examination were included in the study. Scoliosis (major curve Cobb angle ≥ 10°) was confirmed in all 73 patients, pelvic obliquity (iliac crest height difference > 1 cm or sacral takeoff angle > 5°) appeared in 23 (31.5%) patients with scoliosis, and LLD (> 1 cm femoral head height difference) was identified in 6 (8.2%) patients with scoliosis and pelvic obliquity. At a subsequent visit, a mean of 2.8 (range 2–5.8) years later, no significant change (*p* > 0.05) in limb length inequality was observed but a statistically significant increase (*p* < 0.05) in scoliotic and pelvic deformity parameters was found.

**Conclusions:**

In adolescent patient population with thoracic or thoracolumbar scoliosis, the anisomelia remains stable with growth but both the scoliotic deformity and pelvic obliquity progress.

**Trial registration:**

MGH no 2012-P-000774/1

## Background

Limb length discrepancy (LLD) or anisomelia is known to cause pelvic obliquity in the frontal plane resulting in a lumbar scoliosis that is non-structural and non-progressive [1–6]. Forty to 60% of children with lumbar scoliosis have been shown to have pelvic obliquity as well [7, 8]. This pelvic obliquity may be due to LLD, and both pelvic obliquity and scoliosis have been shown to regress with the equalization of LLD [9]. The incidence of LLD has been found to range from 3 to 15% in the general population [10] with the incidence of idiopathic scoliosis approximately 1.5–2% [11].

We have seen many children presenting for the first time in a pediatric scoliosis clinic with LLD in addition to scoliosis. To our knowledge, there are no studies in the literature regarding the association of LLD and pelvic obliquity in adolescence with thoracic or thoracolumbar scoliosis.

In the present study, it is aimed to define if there is a quantitative association between pelvic obliquity, LLD, and the scoliotic curve in an adolescent pediatric population initially presenting for scoliosis evaluation in a pediatric clinic and to evaluate the progression of the scoliotic curve in relation to the different amounts of leg length discrepancy. Our hypothesis was that scoliotic curve increases more with growth in adolescents with LLD rather than in adolescents without LLD.

## Methods

Following institutional review board approval (no. 2012-P-000774/1, date May 1, 2012), a retrospective radiographic and clinical review of children who initially presented to a busy urban pediatric orthopedic service with a diagnosis of idiopathic scoliosis was conducted. Patients were followed for a minimum of 2 years to determine if there was a difference in the magnitude of the major scoliotic curve or LLD.

**Fig. 1:**
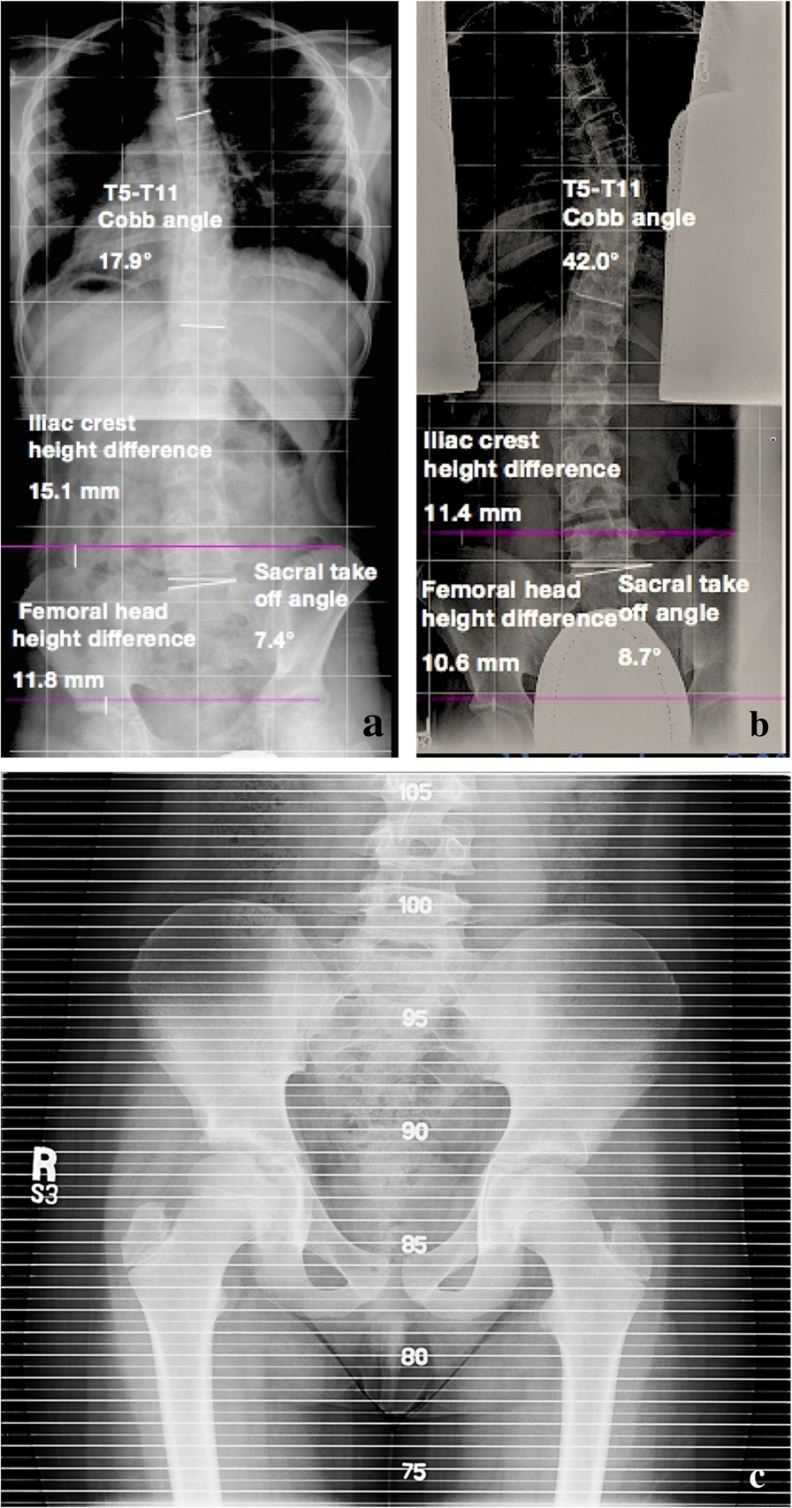
A 10-year-old girl first presented **a** with a major scoliotic curve between T5-T11 of 18°, sacral takeoff angle of 7°, 15-mm iliac crest height, and 12-mm femoral head height difference. At that time, she was treated initially by observation (and later by brace). More than 2 years later, **b** the curve Cobb angle increased to 42°, sacral takeoff angle decreased to 9°, iliac crest height difference decreased to 11 mm, and femoral head height difference decreased to 11 mm. **c** Pelvis view of lower limb scanogram of the same patient obtained at the time of first examination with leg length discrepancy of 11 mm, right longer than left

Patients were included if they were adolescents (between 10 and 16 years old for girls and 10 and 18 years old for boys) with incomplete vertebral growth (with Risser ≤ 4) and had full-length spine PA standing radiographs with unshielded hips. Radiographs had to be obtained out of any brace, and patients had to have been seen at least twice with a minimum of 2 years between successive visits (Fig. 1a, b). When there were radiographic exams at multiple time points within adolescence, the earliest and the latest X-rays were chosen. Exclusion criteria included patients with the apex of major scoliotic curves in the lumbar area (at or below disc L1-L2), Risser sign of 5, syndromic or congenital spinal or lower extremity deformities, metabolic bone diseases or tumors, post-traumatic spine or lower extremity conditions, neuromuscular or physeal plate disorders, and previous spine or extremity surgery or had surgery for scoliosis before the second follow-up. Data collected included demographics (age, height, weight, gender, date/age of menarche), clinical intervention (clinical measurement of limb length inequality, type of treatment, i.e., observation, bracing, shoe lift), and radiographic parameters (magnitude of major scoliotic curve, Risser sign, Nash/Moe rotation of the apical vertebra, sacral takeoff angle [angle between the upper sacral endplate with the horizontal level] as well as iliac crest height difference [distance of most proximal iliac crest points bilaterally] in the coronal plane as measures of pelvic obliquity, and femoral head height difference [distance of most proximal femoral head points bilaterally] as a measure of anisomelia). The change (value at time 2 minus value at time 1) of major curve Cobb angle, femoral head height difference, iliac height difference, and sacral takeoff angle was calculated. Scoliosis was defined as a coronal Cobb angle of ≥ 10°, LLD measured by femoral head height difference > 10 mm, and pelvic obliquity measured by sacral takeoff angle > 5° or iliac crest height difference > 10 mm.

The criteria for brace treatment were Risser 0–2, primary curve angles 25–40°, no prior treatment, and, if female, either premenarchal or less than 1 year postmenarchal. The criterion for heel lift insertion in the shoe of the shorter limb was coronal decompensation with tendency to fall with the scoliosis brace on.

Radiographic measurements were performed twice via the local CAS medical system (Clinical Application Suite Medicity, Salt Lake City, USA), and the average was used in the primary analysis. Magnification and calibration of images were used for accurate measuring, and decimal numbers of values (as angle degrees or millimeters) were rounded in the most proximal integer. All radiographs were digital, and it was ensured that patients were completely erect with the hips and knees extended and their pelvis was not rotated. An effort was made to ensure that the whole pelvis was visible on the digital radiographs but at least the highest points of femoral heads and iliac crests should be clear. Clinical measurement of limb length inequality was documented in cases with radiological femoral head height difference > 10 mm (so that a clinical verification of radiological measurement exists) and confirmed by a lower limb scanogram.

The intraclass correlation coefficient (ICC) was calculated to determine interobserver and intraobserver reliability as described by Winer [12]. The ICC was used to summarize the overall accuracy of the measurement process relative to variations among subjects in each category. The interobserver and intraobserver ICC reported was calculated from the first observation data. Reliability statistics are presented with a 95% confidence interval (95% CI).

### Statistical analysis

Statistical analysis was conducted using SPSS 10.0 package (Chicago, IL). The incidence of LLD, if any, was calculated. Numerical data were presented as mean (range or SD). Mann-Whitney *U* analysis was applied to determine factors (demographic, clinical, and radiographic) related to the two groups of femoral head height difference (≤ 10 mm, > 10 mm). Wilcoxon signed rank test for paired samples was performed to detect significant changes of parameters studied between the two time points. Statistical differences between groups of major scoliotic curve Cobb angle change and femoral head height difference or use of bracing were detected by chi-square test. Spearman correlation test was used to define correlations between femoral head height difference or magnitude of scoliotic curve progression and radiographic indices (magnitude of major scoliotic curve, Nash/Moe rotation of the apical vertebra, sacral takeoff angle, iliac crest height difference), maturation indices (years to time of presentation from menarche for females, Risser sign), or the treatment mode. Reliability analysis based on alpha model was used to calculate the ICC for interobserver reliability. All statistical tests were conducted at a 0.05 significance level (*p* value).

## Results

**Fig. 2:**
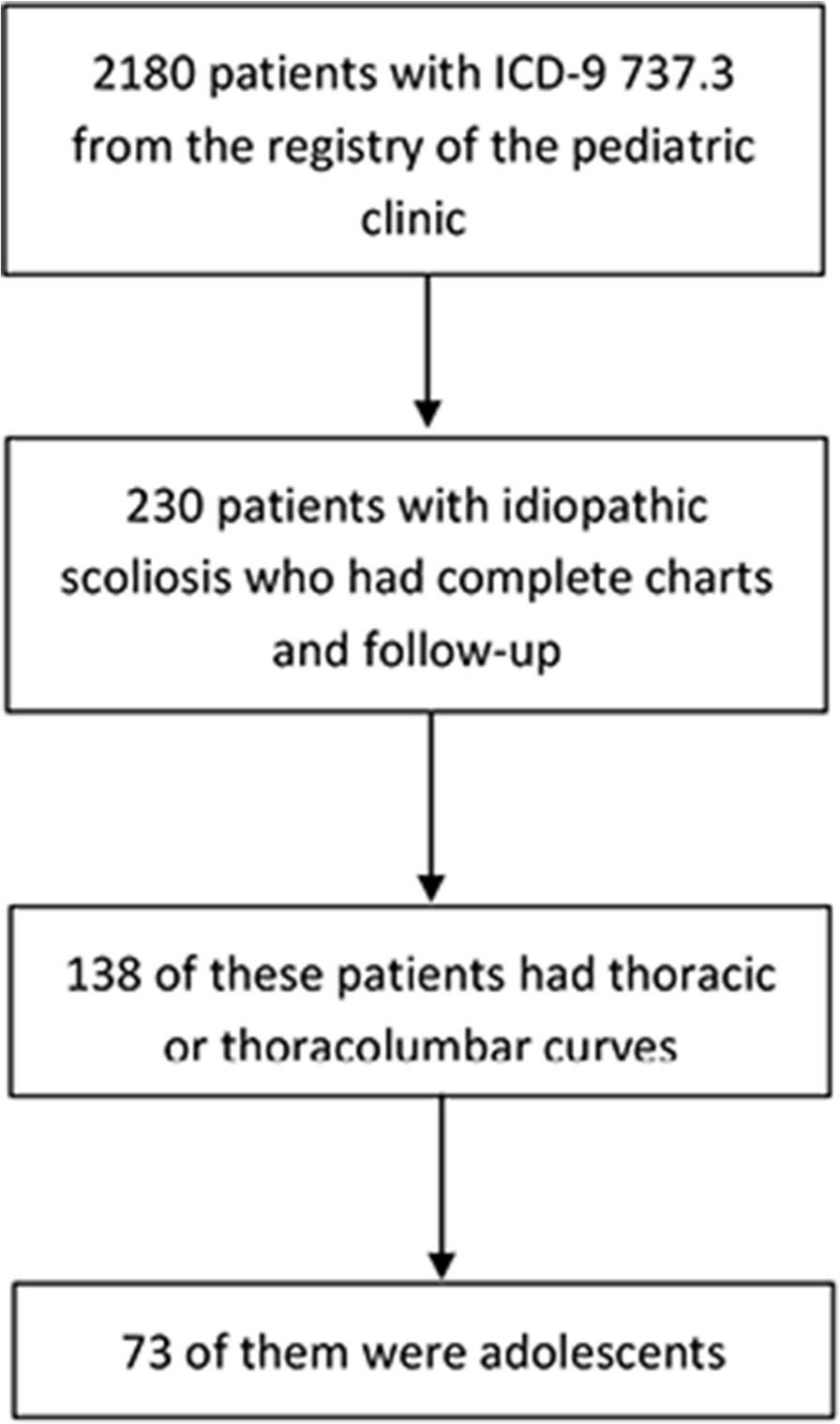
Flowchart showing the selection of the patients who were included in the study

Seventy-three consecutive adolescent patients (24 boys/49 girls) fulfilled the criteria (Fig. 2) and were included in the study. The mean (range) time interval between the two studied visits was 2.8 (2–5.8) years, and the results of the measurements at the two different time points are shown in Table 1. Approximately 3/4 of the patients had observation as treatment after their initial visit while only 1 patient was fit with a heel lift (Table 2).

**Table 1:** Mean (range) of demographic and deformity parameters at different time points

Parameters	Time point 1	Time point 2	*p* value
Age (years)	13.3 (10.3–17.8)	16.1 (12.5–22)	*< 0.001*
Time from menarche (females only) (years)	0.3 (− 3.5–5.9)	3.2 (− 0.1–8.7)	*< 0.001*
Height (centimeter)	153.7(116.5–186)	162.4(136–187)	*< 0.001*
Weight (pound)	115.5(60–310)	138.7(76–321)	*< 0.001*
Body mass index (index)	21.6 (14.9–46)	23.1 (15.6–41.7)	*< 0.005*
Risser sign (index)	2 (0–4)	4.1 (0–5)	*< 0.001*
Major curve Cobb angle (degree)	21.6 (2–42)	26.3 (2–49)	*< 0.001*
Nash/Moe (index) of the apex vertebra	1.3 (1–4)	1.6 (1–4)	*< 0.001*
Iliac crest height difference (millimeter)	5.2 (0–17)	6 (0–20)	*= 0.05*
Sacral takeoff angle (degree)	3 (0–13)	3.4 (0–17)	*< 0.05*
Femoral head height difference (millimeter)	3.8 (0–14)	4.1 (0–16)	= 0.41

*p*-values in italics show statistically significant results

**Table 2:** Type of treatment at time interval between times 1 and 2

Type of treatment	Number of patients	Percentage
Observation	56	76.7
Brace	16 (of which 5 with LLD > 10 mm)	21.9
Heel lift and brace	1 (with LLD > 10 mm)	1.4

At the initial visit, all 73 patients were diagnosed with thoracic or thoracolumbar scoliosis (major curve Cobb angle > 10°). There were no double major curves (both thoracic and thoracolumbar structural curves). The apex of the major scoliotic curve resided in the thoracic (apex cranial to T12-L1, *n* = 51, 70%) and thoracolumbar (apex at T12-L1, *n* = 32, 30%) region, but there was no statistical difference between these two groups (thoracic, thoracolumbar) in all pelvic or femoral head height difference measurements at times 1 and 2 except for the greater change of sacral takeoff angle (*p* < 0.05) at both time points 1 and 2 in the thoracolumbar curve group. Right apex curvatures represented 64.4% (most of them typically thoracic) while 35.6% were left-sided (most of them typically thoracolumbar). The right side of the ilium and femoral head was higher in 54.8% of the cases while the left side was higher in 45.2%. The direction of the major curve apex and the side of the higher iliac wing-femoral head did not correlate (*p* < 0.05).

At the time of initial presentation, iliac height difference of ≤ 10 mm was measured in 60 (82.2%) patients while differences > 10 mm were observed in 13 (17.8%) patients. Sacral takeoff angle of ≥ 5° was seen in 16 (21.9%) patients. Pelvic obliquity (iliac height difference > 1 cm or sacral takeoff angle > 5°) was seen in 23 (31.5%) patients. Also, there were 67 (91.8%) patients with femoral head height difference ≤ 10 mm and 6 (8.2%) with a difference > 10 mm, all 73 patients with idiopathic types of LLD. There were 4 cases with femoral head height difference > 10 mm with typical right-sided thoracic scoliotic curves and 2 cases with femoral head height difference > 10 mm with thoracolumbar curve (1 case with typical left-sided curve and 1 case with atypical right-sided curve but negative MRI for intraspinal disorder). All these 6 patients with LLD > 10 mm had pelvic obliquity and scoliosis. All 6 were treated with a brace, and only one with LLD of 20 mm was treated with a heel lift too due to coronal decompensation and tendency to fall with the scoliosis brace on.

In accordance with the aforementioned two groups of femoral head height difference patients (≤ 10 mm and > 10 mm), there were significant differences (*p* < 0.005) among means of femoral height difference at times 1 and 2, iliac height difference at times 1 and 2, sacral takeoff angle at time 1, and major curve magnitude at time point 2 (Table 3). However, there was no significant difference (*p* > 0.05) in the age at time of presentation (time 1) between these two groups.

**Table 3:** Mean (SD) of spinopelvic parameters in patient groups according to femoral head height difference at presentation

	Group (*N* = 67) with femoral head height difference ≤ 10 mm at time point 1	Group (*N* = 6) with femoral head height difference > 10 mm at time point 1	*p* value
Major curve Cobb angle at time point 1	21.1 (8.2)	27.7 (7.5)	> 0.05
Major curve Cobb angle at time point 2	25.4 (11.1)	36.0 (6.0)	*< 0.05*
Change of major curve Cobb angle between time points	4.3 (8.3)	8.3 (5.0)	> 0.05
Iliac crest height difference at time point 1	4.4 (3.7)	14.7 (1.8)	*< 0.001*
Iliac crest height difference at time point 2	5.2 (4.5)	14.8 (4.5)	*< 0.001*
Change of iliac crest height difference between time points	0.8 (3.3)	0.2 (4.8)	> 0.05
Sacral takeoff angle at time point 1	2.6 (2.4)	7.8 (5.6)	*< 0.01*
Sacral takeoff angle at time point 2	3.2 (2.8)	6.5 (4.7)	> 0.05
Change of sacral takeoff angle between time points	0.5 (1.7)	− 1.3. (5.3)	> 0.05
Femoral head height difference at time point 1	3.1 (2.8)	11.3 (1.8)	*< 0.001*
Femoral head height difference at time point 2	3.4 (4.0)	11.8 (2.5)	*< 0.001*
Change of femoral head height difference between time points	0.3 (3.1)	0.5 (3.0)	> 0.05

*p*-values in italics show statistically significant results

Even though the femoral head height did not change significantly (*p* > 0.05) at last follow-up of the 73 adolescents, the iliac obliquity, the sacral takeoff angle, and scoliotic curve parameters demonstrated an increase (*p* < 0.05). Nevertheless, the group of non-progressive scoliotic curves (increase of Cobb angle < 5°) consisted of 39 (53.4%) patients while the group of progressive scoliotic curves (increase of Cobb angle ≥ 5°) consisted of 34 (46.6%). There was poor positive correlation between the magnitude of curve progression and change of iliac height difference (*r* = 0.25, *p* < 0.05) or sacral takeoff angle (*r* = 0.23, *p* < 0.05) between the two time points, but there was no statistical correlation (*p* > 0.05) between the amount of curve progression and the change of femoral head height difference within the follow-up time or the treatment mode.

The ICC of all radiographic measurements was 0.99 (0.98–0.99) both for interobserver and intraobserver reliability. The lowest ICC for interobserver reliability was seen in the sacral takeoff angle measurement [0.95 (0.94–0.96)] and the highest in iliac crest height [0.99 (0.98–0.99)] and femoral head height [0.99 (0.98–0.99)] measurements. There was no disagreement > 10% between any sequential measurement.

## Discussion

Scoliosis is often associated with pelvic obliquity and can also coexist with LLD [6, 9, 13–15]. In this study of 73 adolescent patients initially evaluated for idiopathic thoracic or thoracolumbar scoliosis, 31.5% were diagnosed with additional pelvic obliquity (iliac crest height difference > 1 cm or sacral takeoff angle > 5°) and 8.2% had also LLD > 1 cm and pelvic obliquity. Within mean interval time between evaluations of 2.8 years, femoral head height difference did not change statistically significantly while scoliosis and pelvic obliquity increased. The 6 patients with LLD (i.e., femoral head height difference > 10 mm) showed pelvic obliquity as well as a thoracic (*n* = 4) or thoracolumbar (*n* = 2) scoliotic curve.

The earliest reported study of the incidence of scoliosis in the general population was by Shands and Eisberg in 1955 [11] and included an analysis of 50,000 minifilms made for a survey of chest disease in the state of Delaware. They determined that 1.9% of the population > 14 years old had scoliosis ≥ 10° and that 0.5% had scoliosis of ≥ 20°. In that group, there was a female-to-male ratio of 3.5:1. Some pioneers in scoliosis surgery have conducted school screening programs to detect scoliosis [2, 16, 17]. In Greece, the prevalence of scoliosis (defined as a curve of ≥ 10°) was 1.7% (1436 of 82,901 children), and most of the curves (1255; prevalence 1.5%) were small (10 to 19°) [18]. In our study of 73 adolescent pediatric patients evaluated for thoracic or thoracolumbar scoliosis in a tertiary hospital, the average Cobb angle at the initial visit was 22°.

Pelvic obliquity is frequently seen in patients with limb length discrepancies as well as in patients with scoliosis [6, 9, 13–15, 19]. Cummings et al., [5] in a study of relatively healthy women, found that posterior innominate bone rotation occurs on the side of the longer limb and anterior rotation occurs on the side of the shorter limb. Schwender and Denis [7] reported, when studying adolescent idiopathic scoliosis cases with lumbar curves > 40°, that iliac obliquity (present in 60% of the cases) was nearly always seen in the direction of the hemicurve (i.e., the lumbosacral fractional curve below a major lumbar or thoracolumbar curve that begins from L4 and ends in S1 vertebra with accompanied pelvic obliquity). Walker and Dickson [8] screened 5303 schoolchildren aged 10–14 years old for scoliosis. Three hundred seventy-five (7.1%) children had curves of 5–9° inclusive and, of these, 138 (36.8%) had scoliosis secondary to a pelvic tilt. Radiographic measurements showed that the pelvic tilt was due to pelvic difference, leg length inequality, or both, and pelvic difference occurred more commonly in combination with leg length inequality than as an isolated finding. Since many patients did not demonstrate LLD, pelvic obliquity in scoliosis patients could be explained in part from a traction phenomenon created by paraspinal or abdominal muscle tension (chondrodiatasis of iliac apophyses) [4, 7]. In our studied population, patients with major lumbar curves were excluded as such group would have, in great percentage, compensatory pelvic difference and LLD. We found 32% of the adolescents with idiopathic scoliosis to have a pelvic obliquity (iliac crest height difference > 10 mm or > 5° sacral takeoff angle). In the majority of the patients, the higher side of the pelvis/femoral head was independent to the direction of the major scoliotic curve.

LLD has been observed in 3–15% of the population [10]. LLD may be classified as apparent or true. True LLD is a primary disorder with shortening of one limb compared to the other and can lead to a functional scoliosis which reduces when the LLD is treated. On the other hand, apparent LLD is a secondary phenomenon; it is an apparent discrepancy in leg length due to a primary pelvic or spine disorder, and it improves with treatment of the pelvic and/or spine disorder. Leg length equalization has been supported as a procedure to eliminate scoliosis [9]. Papaioannou et al. [20], in a study of 23 young adults who had had significant untreated limb length inequality, found no relationship between the underlying cause of the anisomelia, its duration, or the severity of the spinal abnormality. In their study group, the scoliosis was minor in patients with discrepancies of < 2.2 cm. On the contrary, measuring the radiographs of 106 consecutive patients in a private chiropractic practice, those with limb length inequality > 6 mm often (53% of the cases) had scoliosis and/or abnormal lordotic curves [14]. In our study group, not all patients with limb length difference of ≤ 10 mm had scoliosis or pelvic obliquity but all patients with LLD > 10 mm had pelvic obliquity and thoracic or thoracolumbar scoliotic curves.

It is widely accepted that curve magnitude, chronologic age, and Risser sign are strong predictors of progression for idiopathic scoliosis [21]. LLD may progress with age especially in the accelerated phases of growth, and there are several ways to predict its progression [1, 22, 23]. Our results demonstrated that 8.2% of pediatric adolescent scoliosis patients have a LLD > 1 cm. This difference did not change significantly after a mean of 2.8 years of follow-up. Hoikka et al. [24] reported that leg length inequality had good correlation with pelvic tilt assessed from the iliac crests, a moderate correlation with the sacral tilt, and a poor correlation with the lumbar scoliotic curve. A similar association was seen in our study. In the group of patients with LLD, the severity of pelvic obliquity indices (iliac height difference and sacral takeoff angle) was significantly more severe than that in the group of patients without LLD but major curve Cobb angle magnitude was similar. However, in the follow-up time, major curve Cobb angle was also significantly different in the two groups.

### Study limitations

This study concurrently assesses the progression and association of idiopathic thoracic or thoracolumbar scoliosis, pelvic obliquity, and LLD in adolescent patients. Scoliosis is a dynamic condition, but for research reasons, radiographs at serial time points may show the stability, regression, or progression of this deformity. Cases with major lumbar curves were excluded as these could be the etiology or the result of pelvic obliquity and limb length difference. A limitation to our study is the indirect measurement (femoral head height difference) of LLD rather than by a direct measurement (by limb scanogram) in all cases (that on the other hand would be unethical as causing increased radiation exposure to this young population). Yoshimoto et al. [25] used a similar lesser trochanteric height difference as an estimate of limb length inequality while others marked the upper border of the femoral heads as indicators of the limb lengths [26]. This method has shown high reliability, as in our study, while clinical evaluation of limb length inequality has shown low reliability in comparison to radiological methods [3, 27, 28]. In our study and for the patients with more than 1-cm limb length inequality, the radiological measurement of LLD (measured as femoral head height difference) was a true representation of the clinical limb length difference. Hamstring tightness that disables the patient from full extension of the joints as well as the radiographer not checking for correct positioning might remain an underlying limitation of the study. However, cases with such severe deformity were not included actually.

Differences of 2–3° in Cobb angle or less than a centimeter in height were found statistically significant, but they may be not clinically significant in everyday practice due to measurement error. Changes of more than 5° in Cobb angle and limb length discrepancy more than 1 cm may be clinically significant. However, the measurements in this study can be considered accurate due to the method used.

## Conclusions

LLD is uncommon in adolescents with idiopathic thoracic or thoracolumbar scoliosis. Unlike the patients with smaller anisomelia, the patients who had LLD of > 10 mm showed always pelvic obliquity and major thoracic or thoracolumbar scoliotic curves. Even though LLD remained stable after at least 2 years of growth, scoliosis and iliac difference progressed despite treatment. The small number (6 out of 73, 8.3%) of patients with LLD may render our conclusions weak and indicate the need for wider sample population. Future research could focus on younger patients less than 10 years with anisomelia to detect early-onset scoliosis prevalence and how it changes with growth and treatment.

## Abbreviations

ICC: Intraclass correlation coefficient
LLD: Limb length discrepancy
PA: Posteroanterior

## References

[1] Anderson M, Green WT, Messner MB (1963). Growth and predictions of growth in the lower extremities. J Bone Joint Surg.

[2] Asher MA (1988). Scoliosis evaluation. Ortho Clin North Am.

[3] Brady RJ, Dean JB, Skinner TM, Gross MT (2003). Limb length inequality: clinical implications for assessment and intervention. J Orthop Sports Phys Ther.

[4] Burwell RG, Aujla RK, Freeman BJ, Dangerfield PH, Cole AA, Kirby AS (2006). Patterns of extra-spinal left-right skeletal asymmetries in adolescent girls with lower spine scoliosis: relative lengthening of the ilium on the curve concavity & of right lower limb segments. Stud Health Technol Inform.

[5] Cummings G, Scholz JP, Barnes K (1993). The effect of imposed leg length difference on pelvic bone symmetry. Spine.

[6] D'Amico M (2002). Scoliosis and leg asymmetries: a reliable approach to assess wedge solutions efficacy. Stud Health Technol Inform.

[7] Schwender JD, Denis F (2000). Coronal plane imbalance in adolescent idiopathic scoliosis with left lumbar curves exceeding 40 degrees: the role of the lumbosacral hemicurve. Spine.

[8] Walker AP, Dickson RA (1984). School screening and pelvic tilt scoliosis. Lancet.

[9] Raczkowski JW, Daniszewska B, Zolynski K (2010). Functional scoliosis caused by leg length discrepancy. Arch Med Sci.

[10] Gurney B (2002). Leg length discrepancy. Gait Posture.

[11] Shands AR, Eisberg HB (1955). The incidence of scoliosis in the state of Delaware; a study of 50,000 minifilms of the chest made during a survey for tuberculosis. J Bone Joint Surg.

[12] Winer B (1971). Statistical principles in experimental design.

[13] Labaziewicz L, Nowakowski A (1996). Scoliosis and postural defect. Chir Narzadow Ruchu Ortop Pol.

[14] Specht DL, De Boer KF (1991). Anatomical leg length inequality, scoliosis and lordotic curve in unselected clinic patients. J Manip Physiol Ther.

[15] Young RS, Andrew PD, Cummings GS (2000). Effect of simulating leg length inequality on pelvic torsion and trunk mobility. Gait Posture.

[16] Dickson RA, Stamper P, Sharp AM, Harker P (1980). School screening for scoliosis: cohort study of clinical course. Br Med J.

[17] Lonstein JE. Screening for spinal deformities in Minnesota schools. Clin Orthop Relat Res. 1977;10:33–42. 598137

[18] Soucacos PN, Soucacos PK, Zacharis KC, Beris AE, Xenakis TA (1997). School-screening for scoliosis. A prospective epidemiological study in northwestern and central Greece. J Bone Joint Surg.

[19] Aaron AD, Eilert RE (1996). Results of the Wagner and Ilizarov methods of limb-lengthening. J Bone Joint Surg.

[20] Papaioannou T, Stokes I, Kenwright J (1982). Scoliosis associated with limb-length inequality. J Bone Joint Surg.

[21] Lonstein JE, Carlson JM (1984). The prediction of curve progression in untreated idiopathic scoliosis during growth. J Bone Joint Surg.

[22] Moseley CF (1977). A straight-line graph for leg-length discrepancies. J Bone Joint Surg.

[23] Paley D, Bhave A, Herzenberg JE, Bowen JR (2000). Multiplier method for predicting limb-length discrepancy. J Bone Joint Surg.

[24] Hoikka V, Ylikoski M, Tallroth K (1989). Leg-length inequality has poor correlation with lumbar scoliosis. A radiological study of 100 patients with chronic low-back pain. Arch Orthop Trauma Surg.

[25] Yoshimoto H, Sato S, Masuda T, Kanno T, Shundo M, Hyakumachi T (2005). Spinopelvic alignment in patients with osteoarthrosis of the hip: a radiographic comparison to patients with low back pain. Spine.

[26] Segev E, Hemo Y, Wientroub S, Ovadia D, Fishkin M, Steinberg DM (2010). Intra- and interobserver reliability analysis of digital radiographic measurements for pediatric orthopedic parameters using a novel PACS integrated computer software program. J Child Orthop.

[27] Clarke GR (1972). Unequal leg length: an accurate method of detection and some clinical results. Rheumatol Phys Med.

[28] Gross MT, Burns CB, Chapman SW, Hudson CJ, Curtis HS, Lehmann JR (1998). Reliability and validity of rigid lift and pelvic leveling device method in assessing functional leg length inequality. J Orthop Sports Phys Ther.

